# *In vitro* Properties of Methanol Extract and Sodium Alginate of *Sargassum polycystum* C. Agardh Brown Seaweed Collected from Malaysia

**DOI:** 10.21315/tlsr2022.33.1.4

**Published:** 2022-03-31

**Authors:** Jamie Mei-Lin Kok, Ching-Lee Wong

**Affiliations:** School of Biosciences, Taylor’s University, Taylor’s Lakeside Campus, No 1, Jalan Taylor’s, 47500 Subang Jaya, Selangor Darul Ehsan, Malaysia

**Keywords:** *Sargassum polycystum*, Methanol Extract, Sodium Alginate, Minerals, Antioxidant, *Sargassum polycystum*, Ekstrak Metanol, Sodium Alginate, Mineral, Antioksidan

## Abstract

The edible brown seaweed, *Sargassum polycystum* C. Agardh was harvested from the coastal region of Malaysia. In this study, analysis of the nutrition and metal content in the methanol extract showed positive for sodium, potassium, magnesium, vitamins A and E and arsenic contamination. The brine shrimp lethality assay (BSLA) revealed the extract to be non-toxic with LC_50_ value of 15.60 mg mL^−1^ (LC_50_ > 1). The antioxidant and antibacterial activities of the methanol extract were measured through various bioassays. The structural and physicochemical characterisation of the NaAlg, analysed through the ^1^H-NMR analysis revealed the M:G ratio of NaAlg at 0.733 with mannuronic (M) and guluronic (G) fractions at *F*_M_ = 0.423 and *F*_G_ = 0.577, respectively. The degraded NaAlg through methods of ultraviolet irradiation and sonication showed an increment in the *in vivo* antioxidant activities at intervals of 15 min, 30 min, 60 min, 90 min and 120 min. The Fourier transform infrared spectroscopy (FTIR) spectra of polysaccharides taken before and after UV irradiation showed breakage of covalent bonds and thus, increase in the intensity of both O–H and C–O stretching vibrations. Therefore, the increment in antioxidant activities observed in the treated samples were related to changes seen in their molecular structures.

## Abstract (fr)

Rumpai laut coklat yang boleh dimakan, *Sargassum polycystum* C. Agardh dituai dari kawasan pantai Malaysia. Dalam kajian ini, analisis kandungan nutrisi dan logam dalam ekstrak metanol menunjukkan ia positif untuk natrium, kalium, magnesium, vitamin A dan E dan pencemaran arsenik. *Brine shrimp lethality assay* (BSLA) mendedahkan ekstrak tersebut tidak bertoksik dengan nilai LC50 15.60 mg mL^−1^ (LC50 > 1). Aktiviti antioksidan dan antibakteria ekstrak metanol diukur melalui pelbagai bioassai. Pencirian struktur dan fizik NaAlg yang dianalisis melalui analisis 1H-NMR, mendedahkan nisbah M:G NaAlg pada 0.733 dengan pecahan manuronik (M) dan guluronik (G) masing-masing pada FM = 0.423 dan FG = 0.577. NaAlg yang dinyahgred melalui kaedah sinaran ultraviolet (UV) dan sonikasi menunjukkan peningkatan dalam aktiviti antioksidan *in vivo* pada selang masa 15 minit, 30 minit, 60 minit, 90 minit dan 120 minit. Spektra FTIR polisakarida yang diambil sebelum dan selepas sinaran UV menunjukkan kerosakan ikatan kovalen dan dengan itu, peningkatan dalam keamatan kedua-dua getaran regangan O–H dan C–O. Oleh itu, peningkatan dalam aktiviti antioksidan yang diperhatikan dalam sampel yang dirawat berkait dengan perubahan yang dilihat dalam struktur molekul.

**Highlight**

- *Sargassum polycystum* methanol extract is rich in primary and secondary metabolites.
- The methanol extract displayed antibacterial action by effectively inhibiting *Bacillus cereus* and *Staphylococcus aureus*.
- Sonication improved the antioxidant activity of sodium alginate, but no structural changes were found using FTIR.

## INTRODUCTION

The brown seaweed, *Sargassum polycystum* is a member of the Phaeophyceae group that dominates the benthic reefs of tropical and temperate region (Hwang *et al.* 2006). There are over 400 species of *Sargassum* distributed in the warm and temperate waters of Indo-western Pacific region, where Malaysia accommodates about 21 species of the *Sargassum* genus (Noiraksar & Ajisaka 2008; Wong & Phang 2004). The intertidal and subtidal *Sargassum* beds in the marine ecosystem are essential in providing food, habitat and nursery grounds for marine organisms while it remains equally important for people who harvest *Sargassum* for food, alginates, feed and also bioactive compounds (Redmond *et al*. 2014).

Traditionally, seaweeds are regarded as a health food in Asian countries and were consumed as a staple item of diet, thus opening opportunities in the development of various seaweed food products, such as noodles, tea, wine and soup (Rupérez 2002). Generally, the mineral content (8% to 40%) and elements found in seaweeds were reported to be higher than that of land plants and animal products (Ito & Hori 1989; Ortega-Calvo *et al.* 1993). Nevertheless, the nutritional contents of seaweed are dependent on the type of seaweed, mineralization methods, environmental factors and the origin of seaweeds (Rupérez 2002). The complex compounds identified in the seaweeds with varying structures and activities from terrestrial plants could be the results of their response to ecological pressure and effective defence mechanism in order to adapt to the harsh environmental conditions in the marine ecosystem (Pérez *et al.* 2016). Approximately 80 of the 400 known *Sargassum* species were screened and analysed for their bioactive metabolites. Pharmacological properties and novel compounds revealed that the major contributors for the therapeutic effects of *Sargassum* is derived from phlorotannins, fucoidans and monoterpenoids. Sodium alginate is generally the main cell wall components of seaweeds with structural functions. Polyunsaturated fatty acids, carotenoids, and other phenolic are among other bioactive compounds that has been identified in brown seaweeds (Pérez *et al.* 2016).

In view to the current demand of seaweeds and the few studies on edible *S. polycystum* from Malaysia, this paper provides an overview of the biochemical, mineral contents and bioactivities of the methanol and sodium alginate extracts. Thus, the information obtained from this current study would add economic value to marine seaweeds of this part of the world.

## MATERIALS AND METHODS

### Sample Collection and Extraction

The *S. polycystum* sample was collected from Teluk Kemang, Port Dickson, Malaysia and rinsed with 0.1% sodium chloride. The sample was pulverized and macerated in methanol (1:10; w.v^−1^) (EMSURE, USA) followed by agitation at 200 rpm in room temperature for 12 h to 16 h. The solvent was evaporated to dryness using a vacuum rotary evaporator.

### Phytochemical Analysis

The presence of terpenoids, cardiac glycosides, flavonoids, saponins, alkaloids and tannins in the crude methanol extract were analysed qualitatively based on the methods of Harbone (1973) and Trease and Evans (1983).

### Antioxidant Assays

The DPPH, FRAP and ABTS assays were performed according to methods described by Rajauria *et al*. (2010), Benzie and Strain (1996) and Boonchum *et al*. (2011), respectively. Briefly, 600 μL of 0.16 mM DPPH^•^ solution was added to 400 μL of extract at different concentrations (10.00 to0.31 mg mL^−1^) and incubated in the dark at 37°C for 30 min and the absorbance of the mixture was read at 540 nm.

The ABTS assay was performed by preparing stock solutions of 14 mM ABTS^•+^ and 4.9 mM potassium persulfate. The working solution was prepared fresh at every run and it consists of equal volumes of the respective stock solution. The mixture was allowed to react in the dark for 12 h to 16 h at room temperature and the absorbance was adjusted to 0.70 ± 0.02 nm at 734 nm. In the cuvette, 700 μL extracts were allowed to react with 300 μL of the ABTS^•+^ solution for 10 min in the dark at 37°C and absorbance was measured at OD_734_ nm.

The extract concentration value was plotted vs. % of inhibition of free radicals and the EC_50_ value was obtained by linear regression. The capacity of the extract to scavenge the DPPH and ABTS radicals were calculated using the following equation:


$$\text{RSA }(\%)=[({\text{A}}_{\text{B}}-{\text{A}}_{\text{E}})/{\text{A}}_{\text{B}}]\times 100$$

The ability of the sample to reduce ferric ion (Fe^3+^) to ferrous ion (Fe^2+^) was measured through the FRAP assay. The stock solutions included, 300 mM acetate buffer, pH 3.6; 10 mM TPTZ (2, 4, 6-tripyridyl-s-triazine) in 40 mM HCl and 20 mM FeCl_3_·6H_2_O. The fresh working solution prepared by mixing the solution in the ratio 10:1:1 (v:v:v) of stock chemicals and preheated at 37°C before use. Briefly, 600 μL of the FRAP reagent was mixed with 400 μL of the samples (10.00 to 0.31 mg mL^−1^) and incubated in the dark for 30 min at room temperature. Equal volumes of sample and FRAP reagent was allowed to react in the dark and measured at OD_593_ nm. The ability of the extract to reduce Fe^3+^ to Fe^2+^ was expressed as mM Fe(II) g^1^ using a calibration curve of FeSO_4_ (0.1 to1.0 mM).

### Total Phenolic Content (TPC)

The TPC was analysed according to Devi *et al*. (2008) with some modification using Folin-Ciocalteu’s method with Gallic acid as a standard. An aliquot of 100 μL samples (10.00 to 0.31 mg mL^−1^) was mixed with 400 μL of 2% Na_2_CO_3_ and incubated for 2 min at room temperature. Five hundred microliter of 50% Folin-Ciocalteau’s phenol reagent was added into the mixture and allowed to stand in the dark for 30 min at room temperature. After incubation, the absorbance was measured at 720 nm. The TPC was expressed as mg Gallic acid equivalent, GAE g^−1^ extract.

### Thin Layer Chromatography

The profile of pigments present in the methanol extract was accessed with the silica-TLC plate following the methods reported by Sathya *et al*. (2017) with modifications. The pre-coated TLC plates (20 cm × 20 cm); 0.25 mm thickness silica gel 60 F_254_ aluminium plates (Merck) were spotted with the crude extract and subjected to the following mobile phase: hexane:ethyl acetate (3:1; v.v^−1^). The blue spot visualised by spraying the plates with a mixture of 1% potassium ferricyanide and 1% ferric chloride and yellow spot with exposure to DPPH^•^ reagent is indicative of their antioxidant properties.

Based on the methods by Rajauria and Abu-Ghannam (2013), the antioxidant activities of fractions separated on the TLC can be quantified with the antioxidant assays. Firstly, the coloured bands on TLC were scrapped and dissolved separately in methanol and centrifuged at 10,000 rpm for 15 min. Subsequently, the supernatant was collected and evaporated to dryness before subjecting to FRAP and DPPH antioxidant assays.

### Analysis of Ash Content

The analysis of the ash content follows the method described by Pearson (1976). The air-dried crude sample was placed in a silica crucible and kept in a muffle furnace and ignited gradually by increasing the temperature up to 500°C to 600°C. The ash was cooled in a desiccator for 30 min and weighed. The total ash content was expressed as g 100 mL^−1^ of the sample.

### Determination of Minerals and Metal Elements

The presence of minerals (calcium, sodium, magnesium and potassium) and metal (arsenic, lead, mercury and cadmium) elements in the dry ash samples were determined according to the modified method as described in the U.S. Environmental Protection Agency (EPA) Revision 2, 1996 and Pharmacopoeia (2013), respectively. The concentration of the minerals was determined using inductively coupled plasma mass spectrometry (ICP-MS) and metal elements with inductively coupled plasma optical emission spectroscopy (ICP-OES).

### Fat-soluble Vitamins

The XDB-C18 column was used (5 μm, 4.6 × 150 mm), the solvent was 90% acetonitrile and 10% methanol, and UV detection was recorded at 324 nm for vitamin A and the fluorescence for vitamin E was detected at Ex λ = 325 and Em λ = 480. Both vitamin A (retinyl palmitate) and E (α-tocopherol) were identified in the extract by comparing their retention times with those of authentic standards. The concentrations of the fat-soluble vitamins were calculated from the integrated areas of the sample and the corresponding standards.

### Antibacterial Assays

The bacteria strains were purchased from the American Type Culture Collection (ATCC) USA; *Escherichia coli* (ATCC 25922), *E. coli* (ATCC 35218), *Bacillus subtilis* (ATCC 6633), *Bacillus cereus* (ATCC 11778), *Staphylococcus aureus* (ATCC 6538), *S. aureus* (ATCC 6854), *Listeria monocytogenes* (ATCC 7646) and *Pseudomonas aeruginosa* (ATCC 27853). The *S. aureus, B. subtilis*, and *E. coli* were cultured on Muller-Hinton Agar (MHA) and *P. aeruginosa* and *L. monocytogenes* on nutrient agar (NA) under aerobic condition for 12 h to18 h at 37°C. The minimal inhibitory concentration (MIC) values were determined using a standard susceptible agar dilution method according to the Clinical Laboratory Standards Institute (CLSI 2006) guidelines. The density of the inocula required for the assay was adjusted to 0.5 McFarland standard (1 × 10^7^ colony-forming units mL^−1^). The sample was dissolved and diluted with DMSO to obtain a range of concentrations (10.00 to 0.31 mg mL^−1^). Then, 50 μL of bacterial suspension was transferred to each well containing 50 μL of extracts and incubated at 37°C for 12 h to18 h. The MIC was defined as the lowest concentration of the compounds to inhibit the growth of microorganisms detected following the addition of 20 μL of 0.20 μg mL^−1^ iodonitrotetrazolium chloride (INT) indicator.

The lowest concentration of the extract that showed no visible growth after subculture was described as minimum bactericidal concentration (MBC). MBC’s was determined by transferring 10 μL of each medium with no visually detectable bacterial growth in fresh media. The negative control used was DMSO, while the positive control was ampicillin and tetracycline.

### Brine Shrimp Lethality Assay (BSLA)

The dry cysts (*Artemia nauplii*) were hatched in artificial sea water (3.8%) in a container attached to an air pump with regular air flow. The hatching chamber was constantly illuminated and incubated for 36 h at room temperature (22°C to 29°C) to obtain the active nauplii. Then, 10 to 15 nauplii were transferred into the 16-well plates, separately containing methanol extracts at different concentrations (10.00 to 0.63 mg mL^−1^). After 24 h of incubation, the numbers of living nauplii were examined and counted. The lethal concentration required to kill 50% of the nauplii (LC_50_) with 95% confidence intervals was calculated by Finney’s table. The median lethal concentration (LC_50_) for the test samples has been obtained by a plot of the percentage of the shrimps killed against the logarithm of the sample concentration and the toxicity classification was based on the study by Carballo *et al*. (2002).

### Extraction of Sodium Alginate

The NaAlg was extracted from *S. polycystum* following the method by Fertah *et al*. (2017) with minor modifications. The powdered seaweed was immersed in 0.2M hydrochloric acid at room temperature for 24 h. Subsequently, the residues were rinsed with distilled water and agitation for 5 h with 2% sodium carbonate. The extracts were filtered and later precipitated with ethanol. The yield of the crude NaAlg samples was dried overnight at 60°C and expressed as a percentage (%) of dry weight (DW).

### Degradation of NaAlg

To prepare the degraded NaAlg, the polymer samples were subjected to UV-irradiation and sonication. The ultraviolet-C (UV-C) irradiation was performed in the UV cabinet at 254 nm monochromatic output. The 1% NaAlg solution was placed in a petri dish and exposed to the UV lamp at time intervals of 15 min, 30 min, 60 min, 90 min and 120 min with constant mixing at every 15 min intervals.

The polysaccharide was subjected to ultrasonic waves (Elma Elmasonic S30H) at 28 kHz frequency, 1 cm above the transducer. Ten millilitres NaAlg samples were treated for 15 min, 30 min, 60 min, 90 min and 120 min at 37°C water bath with stirring at regular intervals.

### Chemical Characterisation

Infrared spectra of the polysaccharide samples analysed with a Fourier transform infrared spectroscopy (FTIR) spectrophotometer (Perkin-Elmer Spectrum^™^ 1000) equipped with attenuated total reflection accessory (ATR) containing a diamond/ZeSe crystal. The NaAlg was dried in the oven at 60°C before the analysis. The ATR-FTIR spectra were recorded in the 650 to 4000 cm^−1^ range using 16 scans and resolution of 4 cm^−1^.

The NaAlg samples were subjected to partial hydrolysis with acid reflux following the methods described by Jensen *et al.* (2015). The NaAlg solution (0.1%) was prepared with Milli-Q water. The pH of the solution was adjusted to pH 5.6 using HCl (1 and 0.1M) and reflux at 100°C for 1 h. After cooling at room temperature, the pH was adjusted to pH 3.8 and reflux for an additional 30 min. Then, the sample was cooled in ice to stop the hydrolysis, neutralised to pH 7 using NaOH (1 and 0.1UV-CM) and freeze-dried. The ^1^H NMR spectra were acquired on 0.1% w/v solutions of NaAlg in D_2_O with a Fourier-transform Bruker 250 BioSpin supplied with an inverse multinuclear gradient probe-head with z-shielded gradient coils, and with a silicon graphics workstation, at different temperatures.

Following Grasdalen *et al.* (1979) method, the composition and the block structure of alginate can be quantitatively derived from the following relationships:


$$F_{\text{G}}=A_{\text{A}}/(A_{\text{B}}+A_{\text{C}});F_{\text{GG}}={\text{A}}_{\text{C}}/(A_{\text{B}}+A_{\text{C}})$$

The mole fraction of M (*F*_M_) was derived from the normalisation condition:


$$F_{\text{G}}+F_{\text{M}}=1.0$$

The relations between the mole fractions and the doublet frequencies are given by:


$$F_{\text{GG}}+F_{\text{GM}}=F_{\text{G}};F_{\text{MM}}+F_{\text{MG}}=F_{\text{M}}$$

### Statistical Analysis

Statistical analyses performed by IBM^®^ SPSS 21.0 statistical package (Chicago, IL, USA). The data were tested for normality (Shapiro-Wilk’s test) and subjected to the test of homogeneity (Levene’s test). The data presented as mean ± standard deviation (SD) and the differences compared by analysis of variance (one-way ANOVA). A posthoc test (Turkey’s test) analysis performed for any significant differences found between the groups (*p*-value < 0.05).

## RESULTS

### Yield of Extract and Phytochemical Analysis

The yield of the methanol extract was 0.57% of DW with the extract tested positive for the presence of tannins, flavonoids, terpenoids and cardiac glycosides.

### Antibacterial Activities

The antibacterial activities of the crude methanol extract of the *S. polycystum* was determined against ten bacterial strains which is reported in Table 1. The extract was most effective against Gram positive bacteria; *S. aureus*, *B. cereus* and *B. substilis* with MIC in the range of 0.31 ± 0.07 to 10.00 ± 0.00 mg mL^−1^ and MBC in the range of 0.63 ± 0.15 to 10.00 ± 0.00 mg mL^−1^. The extract effectively inhibited *E. coli* and *P. aeruginosa*, however, showed no bactericidal activities towards those Gram negative strains.

### Free Radical Scavenging Ability and TPC

Based on the results obtained from Table 1, the *S. polycystum* methanol extract was effective in scavenging the DPPH and ABTS radicals with EC_50_ values of 2.753 and 0.061 mg mL^−1^, respectively. The ABTS radical scavenging activities (55.56% to 88.01%) were higher compared to the DPPH (23.49% to 77.57%). In the FRAP assay, the reducing capacity of the extract was evaluated. Antioxidant activity was expressed as equivalents of mM Fe (II) g^−1^ extract calculated through the Fe_2_SO_4_ linear regression curve with the FRAP value reported for 10 mg mL^−1^ extract at 1.527 ± 0.057 mM Fe (II) g^−1^. Based on the Folin Ciocalteau method, the TPC in the methanol extract (10.00 to 0.31 mg mL^−1^) was calculated in the range of 0.269 ± 0.032 to 85.026 ± 1.078 as mg GAE g^−1^.

### Pigments and Carotenoids

Methanol extract was subjected to TLC fractionation in triplicates (*n* = 3) through the silica chromatography plate. The retention factor (R_f_) for the corresponding fractions was detected and shown in Table 2. The chromatographic profile of the crude extract resulted in 12 distinctive bands. The TLC profiling showed a strong yellow-orange band (F2: R_f_ = 0.056) that corresponded to the fucoxanthin (standard). Among other pigments reported in brown seaweed were chlorophyll *a* and chlorophyll *c*, β-carotene and other xanthophylls. Thus, the orange, yellow, and green bands spotted in the TLC would correspond to some of these pigments. The TLC showed a yellowish-green band (F3: R_f_ = 0.150), bluish-green bands (F6: R_f_ = 0.300; F7: R_f_ = 0.313), greyish-green bands (F8: R_f_ = 0.481, F9: R_f_ = 0.544), reddish-orange bands (F10: R_f_ = 0.638; F11: R_f_ = 0.856) and yellowish band (F12: R_f_ = 0.950).

Moreover, the antioxidant activity of the separated compounds on the TLC plate was accessed qualitatively by treatment with FeCl_3_-FeCN_3_ and DPPH solution (Table 2). Treatment with FeCl_3_-FeCN_3_ showed one distinctive blue spot at F2 (R_f_ = 0.056) which indicated that fucoxanthin has the highest ferric reducing ability. Moreover, F2 showed the highest FRAP value at 53.210 ± 6.870 mg Fe(II) g^−1^ extract. The analysis of the remaining bands (F3 to F12) exhibited lower ferric reducing ability at values ranging from 32.352 ± 2.087 to 45.330 ± 0.963 mg Fe(II) g^−1^ extract. In the analysis of TLC with DPPH assay, all the bands appeared yellow after treatment. When quantified, the fractions showed radical scavenging activity with values in the range of 2.889 ± 1.192 to 7.695 ± 0.399 mg AAE g^−1^ extract.

### Toxicity Assay

After 24 h of exposure, the results of the BSLA with varying concentrations of *S. polycystum* methanol extracts were described in Table 1. The toxicity of the extract was highest at 10 mg mL^−1^ with 90% mortality of nauplii and 0.63 mg mL^−1^ was the lowest concentration with no mortality. The LC_50_ value of the methanol extract was calculated at 15.60 mg mL^−1^ and was therefore considered not toxic on the basis that the LC_50_ > 1 mg mL^−1^.

### Chemical Composition

Table 3 shows the total ash alongside some minerals and metal elements in *S. polycystum* methanol extract. Ash content was reported at 0.06 mg L^−1^ and among the minerals tested, Na and K were in highest concentration at 9.87 and 5.52 mg 100 mL^−1^, respectively. Even though the heavy metal contaminant As (arsenic) was present in the extract at 0.17 mg L^−1^, nevertheless, metals such as Pb, Hg and Cd were below the detectable limits ( < 0.01 mg L^−1^). The identification and quantification of vitamins were performed by reversed-phase HPLC. The peak of vitamin A and vitamin E were identified by comparing their retention time with the standards; retinyl palmitate (3.16 min) and tocopherol (13.03 min). The concentration of the vitamins were calculated as 1.21 mg L^−1^ and 1.79 mg L^−1^, respectively.

### Yield and Phytochemicals in NaAlg

Ten grams of brown seaweed, *S. polycystum*, yielded 22.51% NaAlg. The phytochemicals detected in the crude NaAlg were saponins and flavonoids. Tannins, terpenoids, cardiac glycosides and phenolics were absent in the extract.

### FTIR and NMR Profile of NaAlg

**Figure 1:**
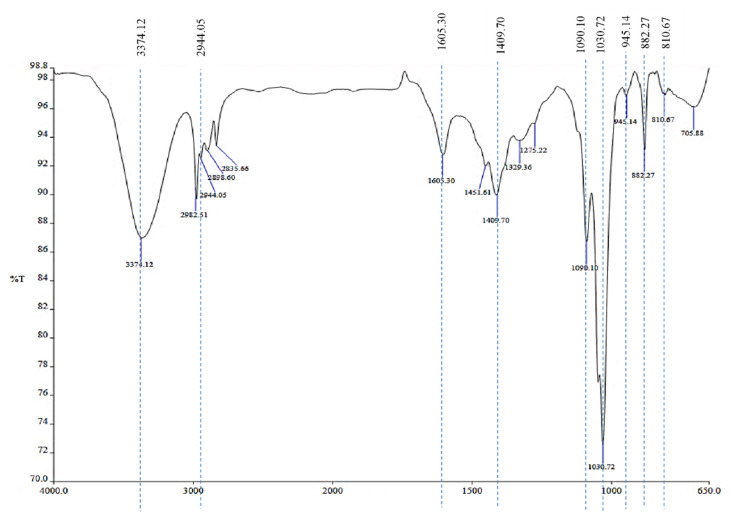
FTIR profile of sodium alginate extracted from *S. polycystum.*

The spectrum for NaAlg extracted from *S. polycystum* was in agreement with the finding by Fertah *et al*. (2017) and Fenoradosoa *et al*. (2010). The ATR-FTIR spectrum of NaAlg extracted from *S. polycystum* with functional groups that corresponded to the absorption bands in 4000 to 650 cm^−1^ range is depicted in Fig. 1. The broad peak that appeared at 3374.12 cm^−1^ in the range of 3,000 to 3600 cm^−1^ was assigned to stretching vibration of the hydrogen bonded O–H and a weak signal at 2944.05 cm^−1^ was assigned to stretching vibrations of C–H. The band at 1605.30 cm^−1^ and 1409.70 cm^−1^ corresponds to the asymmetrical and symmetrical stretching of carboxylate group, respectively. Peaks at 1090.10 cm^−1^ and 1030.72 cm^−1^ were associated with the stretching of the C–O group of pyranose rings and the band at 945.5 cm^−1^ was indicative of the C–O stretching vibration of uronic acid. The band at 810.67 cm^−1^ can be assigned to the characteristic of mannuronic acid residues.

**Figure 2:**
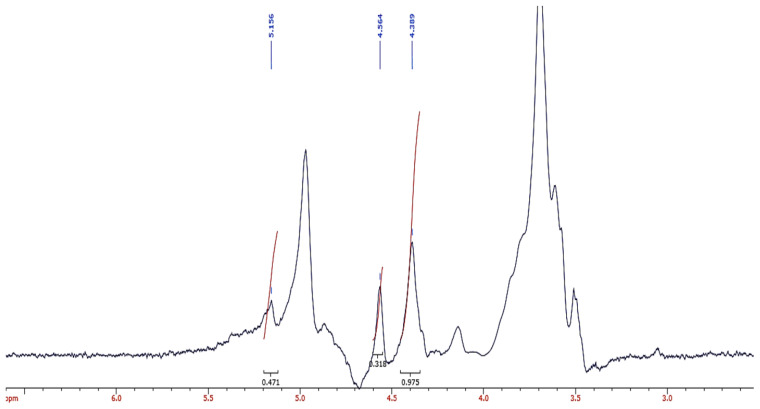
^1^H NMR spectrum of sodium alginate extracted from *S. polycystum.*

The ^1^H NMR spectroscopy is suitable for characterising both the composition and the distribution sequence of the two uronate residues in alginate samples (Yuan & Macquarrie 2015). In Fig. 2, the ^1^H NMR spectra of NaAlg sample showed specific peaks of 5.16, 4.56, and 4.39 ppm assigned to H1-G (peak A), H1-M + H5-GM (peak B), and H5-GG (peak C), respectively. The composition of the mannuronic and guluroric acid frequencies in *S. polycystum* species were *F*_M_ = 0.423; *F*_G_ = 0.577; *F*_MM_ = 0.337; *F*_GG_ = 0.491; *F*_GM_ = 0.086 and an M/G ratio of 0.733 (M/G < 1).

### Antioxidant Activities of Degraded NaAlg

The antioxidant activities of the treated polysaccharides via UV-irradiation and sonication were reported in Table 4. The *S. polycystum* NaAlg showed higher antioxidant activities compared to the commercial samples and showed an increment in activities after treatment for 15 min to 120 min. Overall, the sonicated samples showed an increase in ABTS radical scavenging activity at (0.73% to 11.71%) compared to the UV irradiated sample (2.43% to 9.19%) with increasing exposure time. Nevertheless, the UV irradiated NaAlg showed a higher increment at 15 mins compared to the sonicated sample. The sonicated samples also showed an increment in reducing ability by 28.31% to 61.72% compared to the UV irradiated samples at 39.12% to 50.92%. Comparatively, DPPH^•^ scavenging activity of UV irradiated *S. polycystum* showed a better increment in activity compared to sonicated samples with 16.37% to 60.99% and 22.19% to 49.16%, respectively.

### FTIR Profile

**Figure 3:**
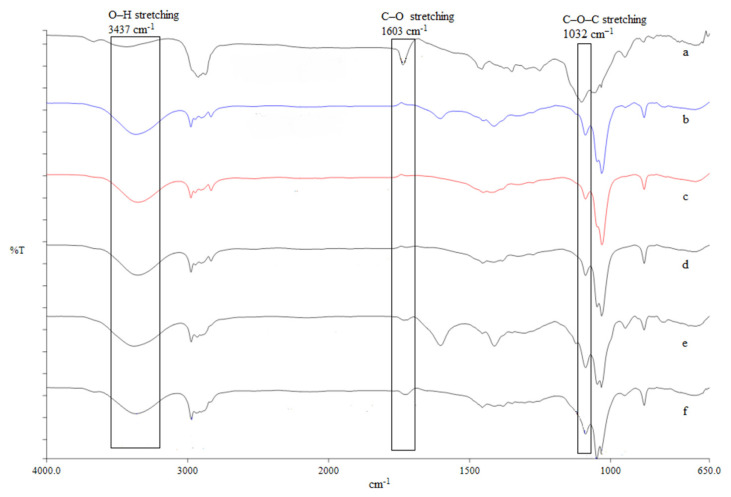
FTIR spectra of *S. polycystum* sodium alginate exposed to UV light: (a) untreated sample, (b) 15 min, (c) 30 min, (d) 60 min, (e) 90 min, (f) 120 min.

The FTIR was used to access the changes in the functional groups of the treated NaAlg samples by sonication and UV-irradiation before and after treatment. In Fig. 3, the UV irradiated NaAlg spectrum for *S. polycystum* showed a noticeable increase in intensity at band 3436 cm^−1^ and 1603 cm^−1^, assigned to the stretching vibrations of hydroxyl and carboxylate groups, respectively. However, the band at 1032 cm^−1^ ascribed to C–O–C groups that showed a decrease in intensity over treatment time. The spectrum profile of the sonicated samples showed no obvious changes at an increasing degradation time, and thus, may indicate that structural changes could have been induced through a different mechanism (Fig. not shown).

## DISCUSSION

In the present study, the *S. polycystum* was isolated from the coastal region, Port Dickson, Malaysia and the crude methanol and sodium alginate extracts were studied on its nutritional content and bioactivities. Among the eight strains investigated, the antibacterial profile of the methanol extract revealed that the Gram positive strains were more susceptible than the Gram-negative strains, *E. coli* (ATCC 25922; ATCC 35218) and *P. aeruginosa* (ATCC 27853) that exhibited MIC but no MBC activities. A similar study by Kausalya and Rao (2015) reported that 100 mg mL^−1^ of the *S. polycystum* methanol extract was effective in inhibiting several Gram negative strains among which is *E. coli*. Even though the extract was not effective against the Gram positive, *L. monocytogenes*, nevertheless they showed inhibitory and bactericidal activities against the other six strains (Table 1). A review by Jaswir (2014) on the antibacterial activity of several *Sargassum* species from Malaysia revealed that the methanol extracts of *S. binderi* and *S. plagyophillum* inhibited both *B. subtilis* and *S. aureus* whereas *S. flavellum* was only effective against *B. subtilis*. However, the methanol extract of the *Sargassum* species studied showed no inhibition against *P. aeruginosa* and *E. coli*. Therefore, given the effectiveness of *S. polycystum* extract against the selected strain among the other *Sargassum* genus thus, further steps of elucidating the potential compounds and their biochemical pathway are worth exploring.

The comprehensive analysis of the antioxidant activities of 10 mg mL^−1^ of the extract showed high DPPH and ABTS radical scavenging activity at 77.57% and 88.01% and EC_50_ values of 2.753 and 0.061 mg mL^−1^, respectively (Table 1). As has been reported in several studies, the antioxidant activities in the *Sargassum* genus has been well documented. Among the methanol extract of the brown seaweed studied by Matanjun *et al.* (2009), *S. polycystum* (FRAP= 366.69 μM mg^−1^ dry extract; TPC = 45.16 mg PGE g^−1^ dry extract) exhibited the highest activities compared to *Dictyota dichotoma* and *Padina* sp. extracts. The Pearson’s correlation analysis showed that there was a positive correlation between TPC with reducing power (*p* < 0.05; R = 0.940), and DPPH radical scavenging activity (R = 0.859). However, no significant correlation between TPC and ABTS (*p* > 0.05; R = 0.757) were observed. The strong correlation between FRAP and TPC suggests that polyphenols such as phlorotannins have the ability to reduce (Fe^3+^-TPTZ) to (Fe^2+^-TPTZ) and thus, have the ability to donate electrons to reduce lipid peroxidation, so that they can act as primary and secondary antioxidants (Matanjun *et al.* 2009; Devi *et al.* 2011). In addition to that, the qualitative TLC analysis revealed F2 spot (fucoxanthin) as the most apparent compound when treated with FeCl_3_-FeCN_3_ and the FRAP value quantified was 53.210 ± 6.870 mg Fe(II) g^−1^ extract. Therefore, the results suggest that polyphenolic compounds and fucoxanthin may have contributed to the antioxidant activity observed in FRAP assay.

The mineral content of the crude methanol extract of *S. polycystum* is shown in Table 3. Generally, a lower ash content was measured at 0.06 mg L^−1^ and thus, explaining the lower content of elements detected in the extract (Na, K and Mg) with the Na:K ratio of 1.79. Even though the Na:K content in this study was slightly higher than recommended by the World Health Organization (WHO) with an optimal Na:K ratio of ~1, nevertheless, the ratio reported in this study was relatively lower than those reported in processed meats, white bread/rolls, and savoury sauces and condiments, with Na:K ratios of 7.8, 6.0, and 5.4, respectively (O’Halloran *et al.* 2016). Besides that, the two trace elements identified was Mg (6.15 mg L^−1^) and heavy metal, As (0.17 mg L^−1^). The level of the As is within the allowed limits by Food and Drug Administration, 2018 for cosmetic (< 10 mg L^−1^), however slightly higher for consumption (< 0.1 mg L^−1^). Besides that, the toxicity study conducted through the BSLA revealed that the methanol extract was non-toxic with LC_50_ of 15.60 mg mL^−1^.

Further analysis of the NaAlg extract was conducted (Table 4). Comparatively, in this study, higher NaAlg (20.00%, DW) yield was reported compared to *S. wightii* (21.71%) and *S. myriocystum* (20.10%) (Subramanian *et al*. (2015). Subsequently, the antioxidant study revealed that NaAlg from *S. polycystum* reported higher antioxidant activities (DPPH = 7.61 ± 2.74 μM TE.g^−1^; FRAP = 34.68 ± 0.23 μM TE.g^−1^, ABTS = 34.08 ± 0.10 μM TE.g^−1^) compared to commercial NaAlg (DPPH = 0.57 ± 2.23 μM TE.g^−1^; FRAP = 4.28 ± 1.03μM TE.g^−1^, ABTS = 8.60 ± 0.10 μM TE.g^−1^). This could be due to the secondary metabolites impurities such saponins and flavonoids detected through the phytochemical analysis. Marimuthu *et al.* (2012) have also reported similar impurities in the NaAlg extract of *S. wightii*. The compounds have a high tolerance level towards various factors such as heat and UV rays, and therefore could be an advantage in various industrial applications as they possess several biological activities such as antimicrobial, antiviral, antioxidant and anticoagulant (Jeyaraman *et al*. 2013; Cox *et al.* 2011). Thus, it is worth exploring the synergistic effects of NaAlg and the impurities present as a powerful antioxidant tool.

The analysis of NaAlg through the FTIR analysis could be used as a method to identify the presence of NaAlg. Fig. 1 shows the characteristic peaks of NaAlg in the range of 950 to 750 cm^−1^ with the peak (945.5 cm^−1^) indicating the C–O is stretching vibration of uronic acid and peak (810.67 cm^−1^) assigned to mannuronic acid residues (Fertah *et al.* 2017). The ^1^H-NMR analysis of the extracted NaAlg shown in Fig. 2 indicated an M:G ratio of 0.733 which was similar to other *Sargassum* species that shows values in the range of 0.94 and 1.07 (Fenoradosoa *et al*. 2010). However, the similar author reported NaAlg from *S. polycystum* with a lower M:G (0.21) compared to the NaAlg in this study. It has been previously reported that such discrepancy was evident for *S. fluitans* and *S. oligocystum* that showed variation in the M:G ratio depending on the extraction procedures and the origin of the seaweed with values ranging from 0.52 to 0.57 and 0.49 to 0.62, respectively (Torres *et al.* 2007).

Further increment in the NaAlg samples was performed via UV-irradiation and sonication methods. Increasing antioxidant activities (DPPH, FRAP and ABTS) of the treated polysaccharides with increasing treatment time of 15 min to 120 min is shown in Table 4. The FTIR profile, in Fig. 3 shows changes in the absorption band at 3,437 cm^−1^ (OH groups) where the intensity broadens with irradiation time. Simultaneously, the decreasing peak intensity of 1,603 cm^−1^ (C–O) and 1,032 cm^−1^ (C–O–C) was perceived consistent with the random scission of the glycosidic bonds of the NaAlg backbone and the formation of new functional groups, carbonyl and carboxyl groups that enhances the controls the antioxidant behaviour of polysaccharide-type polymers (Choi *et al.* 2009; Nagasawa *et al.* 2000). The spectrum analysis of the sonicated NaAlg detected no obvious changes to the functional groups. Wasikiewicz *et al.* (2005) reported similar findings and suggested that changes in the molecular weight of NaAlg from 2.16 × 10^6^ Da to 2.92 × 10^5^ Da via sonication undergoes different mechanism than ultraviolet and gamma degradation.

## CONCLUSIONS

Overall, the methanol extract and NaAlg from brown seaweeds are commonly extracted and explored by industries for various applications, thus, an overview of the bioactivities and physicochemical analysis of the extracts from *S. polycystum* could pave future studies on this species from Malaysia. In this study, the crude methanol extract showed the presence of beneficial elements such as Na, K, Mg, vitamin A and vitamin E. In addition to that, due to its low toxicity, high antioxidant activities and antibacterial activities against food pathogen, the extract could be further developed as dietary supplements, food products or cosmetics. Besides that, the effects of UV-irradiation and sonication could be employed as effective methods of improving the antioxidant activities of NaAlg. Generally, at 120 min, the treatment of polysaccharides under sonication showed a higher increment in antioxidant activities compared to UV-irradiation. However, considering the time required for degradation to take effect, the ultraviolet seems to be the better method compared to ultrasonic degradation. However, limitations such as low UV light penetration and its application to a limited depth of the solution may restrict its commercial applicability. Nevertheless, this method deemed more convenient, easy and cost-effective process for the industry. Besides that degraded polysaccharides are more soluble and less viscous and thus, will not alter the texture and the appearance of the products. Thus, at present, our group is exploring *in vivo*, their cytotoxic activity upon potential application and commercialisation.

## Figures and Tables

**Table 1:** Antibacterial (MIC and MBC), antioxidant (DPPH, FRAP and ABTS), total phenolic content (TPC) and brine shrimp lethality assay (BSLA) of crude methanol extract from *S. polycystum.*

Antibacterial assay				Antioxidant assay						Folin-Ciocalteau assay	Brine Shrimp Lethality assay (BSLA)	
			
Bacteria strain	Gram	MIC	MBC	Concentration	DPPH		ABTS		FRAP	TPC	Mortality	LC_50_
							
	+ / −	mg mL^−1^	mg mL^−1^	mg mL^−1^	RSA %	EC_50_ mg mL^−1^	RSA %	EC_50_ mg mL^−1^	mM Fe(II) g^−1^	mg GAE g^−1^	%	mg mL^−1^
*E. coli* ATCC 25922	−	1.25 ± 0.29	NI	0.310	23.49	2.753	55.56	0.061	0.028 ± 0.001	0.269 ± 0.032	0	15.60
*E. coli* ATCC 35218	−	1.25 ± 0.29	NI	0.630	43.56		76.90		0.161 ± 0.008	4.674 ± 0.038	0	
*P. aeruginosa* ATCC 27853	−	0.31 ± 0.07	NI	1.250	54.11		74.04		0.246 ± 0.030	14.124 ± 0.406	5.00	
*S. aureus* ATCC 6538	+	0.31 ± 0.00	2.50 ± 0.20	2.500	55.41		81.41		0.383 ± 0.100	21.601 ± 0.292	11.00	
*S. aureus* ATCC 6854	+	2.50 ± 0.07	10.00 ± 0.00	5.000	58.98		84.60		0.590 ± 0.443	60.164 ± 1.575	13.00	
*B. cereus* ATCC 11778	+	0.31 ± 0.07	0.63 ± 0.15	10.000	77.57		88.01		1.527 ± 0.057	85.026 ± 1.078	90.00	
*B. substilis* ATCC 6633	+	2.50 ± 0.07	5.00 ± 0.00									
*L. monocytogenes* ATCC 7646	+	NI	NI									

*Notes*: Tests were performed in triplicates (n = 3) and values are expressed as mean ± standard deviation (SD). MIC: minimum inhibitory concentration; MBC: minimum bactericidal concentration; RSA: Radical scavenging acitivity; TPC: total phenolic content

**Table 2:** Separations of methanol crude extract on silica-TLC plate with their respective R_f_ value and antioxidant activities.

Methanol silica-TLC fractions	R_f_ value	Antioxidant Assay		

		DPPH		FRAP
	
		RSA (%)	mg AAE g^−1^ extract mg	Fe(II) g^−1^ extract
1	0.000	32.85	6.223 ± 0.577	28.181 ± 0.700^*^
2	0.056	25.92	4.251 ± 1.730	53.210 ± 6.870^*^
3	0.150	38.02	7.695 ± 0.399	36.709 ± 1.274
4	0.181	36.85	7.362 ± 1.356	34.114 ± 0.850
5	0.269	35.13	6.871 ± 0.544	35.597 ± 0.736
6	0.300	32.71	6.184 ± 1.077	39.119 ± 1.124
7	0.313	28.65	5.027 ± 0.743	43.940 ± 8.022
8	0.481	32.89	6.233 ± 1.358	36.987 ± 1.472
9	0.544	29.89	5.379 ± 0.407	45.330 ± 0.963
10	0.638	21.483	2.989 ± 0.162^*^	36.640 ± 0.579
11	0.856	21.13	2.889 ± 1.192^*^	43.384 ± 0.834
12	0.950	33.20	6.322 ± 0.800	32.352 ± 2.087

*Notes*: Values are expressed as mean ± standard deviation. Values with (*) in columns are significantly different (*p* < 0.05), n = 3. DPPH and FRAP activity, are expressed as mg AAE/g extract and mg Fe(II)/g extract respectively

**Table 3:** The minerals, vitamins and metal elements in *S. polycystum* methanol extract.

Parameter	Concentration
Ash (mg L^−1^)	0.06
pH at 25°C	8.50
Sodium, Na (mg 100 mL^−1^)	9.87
Calcium, Ca (mg 100 mL^−1^)	ND (< 0.10)
Magnesium, Mg (mg L^−1^)	6.15
Potassium, K (mg 100 mL^−1^)	5.52
Vitamin A (mg L^−1^)	1.21
Vitamin E (mg L^−1^)	1.79
Arsenic, As (mg L^−1^)	0.17
Lead, Pb (mg L^−1^)	ND (< 0.01)
Mercury, Hg (mg L^−1^)	ND (< 0.01)
Cadmium, Cd (mg L^−1^)	ND (< 0.01)

*Notes*: The methanol extract of *S. poycystum* was tested at 10 mg. mL^−1^. ND: Not detected; (<) less than the minimum detection limit detected.

**Table 4:** The antioxidant activities of UV irradiated and sonicated sodium alginate at the exposure time of 15 min, 30 min, 60 min, 90 min and 120 min with the percentage (%) increment of the antioxidant activities.

	Time (min)	iAntioxidant Activity of Treated NaAlg (μM TE/g extract)											

		UV						SN					
	
		0	15	30	60	90	120	0	15	30	60	90	120
ABTS	*S. polycystum*	^a^34.08 ± 0.1	^b^34.93 ± 0.07	^c^35.98 ± 0.10	^d^36.34 ± 0.14	^de^37.04 ± 0.10	^e^37.53 ± 0.10	^a^34.08 ± 0.10	^ab^34.33 ± 0.07	^b^35.43 ± 0.10	^c^36.45 ± 0.10	^d^38.11 ± 0.10	38.60 ± 0.14
	Commercial	^a^8.60 ± 0.10	^b^9.72 ± 0.24	^c^11.27 ± 0.14	^d^13.28 ± 0.10	^d^13.97 ± 0.10	^e^15.45 ± 0.10	^a^8.60 ± 0.10	^b^9.92 ± 0.10	^c^11.94 ± 0.10	^d^13.37 ± 0.04	^e^15.11 ± 0.14	^e^16.10 ± 0.10
FRAP	*S. polycystum*	^a^34.68 ± 0.23	^b^38.59 ± 0.10	^c^39.20 ± 0.28	^d^44.80 ± 0.13	^e^45.95 ± 0.19	^f^48.26 ± 0.19	^a^34.68 ± 0.23	^b^42.05 ± 0.00	^c^45.47 ± 1.53	^c^45.97 ± 0.22	^d^46.43 ± 0.75	^e^53.80 ±0.76
	Commercial	^a^4.28 ± 0.10	^b^7.03 ± 0.13	^c^7.47 ± 0.19	^c^7.78 ± 0.16	^d^8.30 ± 0.33	^d^8.72 ± 0.35	^a^4.28 ± 0.10	^b^5.97 ±0.10	^c^6.76 ± 0.53	^d^8.41 ± 0.20	^e^10.72 ± 0.10	^f^11.18 ± 0.07
DPPH	*S. polycystum*	^a^7.61 ± 0.27	^b^9.10 ± 0.43	^c^12.64 ± 0.43	^d^13.81 ± 0.43	^e^15.56± 0.43	^f^19.51 ± 0.43	^a^7.61 ± 0.27	^b^9.78 ± 0.27	^c^11.39 ± 0.27	^d^12.93± 0.27	^e^13.91 ± 0.27	^f^14.97 ± 0.27
	Commercial	^a^0.57 ± 0.22	^b^1.80 ± 0.22	^c^3.36 ± 0.22	^d^4.79 ± 0.22	^e^5.44 ± 0.22	^f^6.38 ± 0.22	^a^0.57 ± 0.22	^b^1.11 ± 0.20	^c^2.78 ± 0.20	^d^3.85 ± 0.20	^e^4.64 ± 0.20	^f^5.48 ± 0.20

	Time (min)	iiIncrement of Antioxidant Activity (%)											

		UV						SN					
	
		15	30	60		90	120	15	30	60		90	120

ABTS	*S. polycystum*	2.43	5.28	6.22		7.99	9.19	0.73	3.81	6.50		10.57	11.71
	Commercial	11.52	23.69	35.24		38.44	44.34	13.31	27.97	35.68		43.08	46.58
FRAP	*S. polycystum*	10.13	11.53	22.59		24.53	28.14	17.53	23.73	24.56		25.31	35.54
	Commercial	39.12	42.70	44.99		48.43	50.92	28.31	36.69	49.11		60.07	61.72
DPPH	*S. polycystum*	16.37	39.79	44.90		51.09	60.99	22.19	33.19	41.14		45.29	49.16
	Commercial	68.33	83.04	88.10		89.52	91.07	48.65	79.50	85.19		87.72	89.60

i a–f: Row wise values with different superscripts of this type in the same treatment group indicate significant differences (*p* < 0.05) according to the Turkey test (n = 3).

*Note*: All the values are mean ± standard deviation (SD).

ii The percentage (%) increment of antioxidant activities of the samples were deduced by comparing the values of treated and untreated samples at different treatment time.

UV: Ultraviolet; SN: Sonication
